# CD83 expressed by macrophages is an important immune checkpoint molecule for the resolution of inflammation

**DOI:** 10.3389/fimmu.2023.1085742

**Published:** 2023-02-15

**Authors:** Katrin Peckert-Maier, Pia Langguth, Astrid Strack, Lena Stich, Petra Mühl-Zürbes, Christine Kuhnt, Christina Drassner, Elisabeth Zinser, Marius Wrage, Jochen Mattner, Alexander Steinkasserer, Dmytro Royzman, Andreas B. Wild

**Affiliations:** ^1^ Department of Immune Modulation, Universitätsklinikum Erlangen, Friedrich–Alexander Universität Erlangen–Nürnberg, Erlangen, Germany; ^2^ Mikrobiologisches Institut - Klinische Mikrobiologie, Immunologie und Hygiene, Universitäts-klinikum Erlangen and Friedrich-Alexander-Universität (FAU) Erlangen-Nürnberg, Erlangen, Germany

**Keywords:** CD83, macrophages, checkpoint molecule, resolution of inflammation, STAT-6, wound healing

## Abstract

Excessive macrophage (Mφ) activation results in chronic inflammatory responses or autoimmune diseases. Therefore, identification of novel immune checkpoints on Mφ, which contribute to resolution of inflammation, is crucial for the development of new therapeutic agents. Herein, we identify CD83 as a marker for IL-4 stimulated pro-resolving alternatively activated Mφ (AAM). Using a conditional KO mouse (cKO), we show that CD83 is important for the phenotype and function of pro-resolving Mφ. CD83-deletion in IL-4 stimulated Mφ results in decreased levels of inhibitory receptors, such as CD200R and MSR-1, which correlates with a reduced phagocytic capacity. In addition, CD83-deficient Mφ upon IL-4 stimulation, show an altered STAT-6 phosphorylation pattern, which is characterized by reduced pSTAT-6 levels and expression of the target gene *Gata3*. Concomitantly, functional studies in IL-4 stimulated CD83 KO Mφ reveal an increased production of pro-inflammatory mediators, such as TNF-α, IL-6, CXCL1 and G-CSF. Furthermore, we show that CD83-deficient Mφ have enhanced capacities to stimulate the proliferation of allo-reactive T cells, which was accompanied by reduced frequencies of Tregs. In addition, we show that CD83 expressed by Mφ is important to limit the inflammatory phase using a full-thickness excision wound healing model, since inflammatory transcripts (e.g. *Cxcl1, Il6*) were increased, whilst resolving transcripts (e.g. *Ym1, Cd200r, Msr-1*) were decreased in wounds at day 3 after wound infliction, which reflects the CD83 resolving function on Mφ also *in vivo*. Consequently, this enhanced inflammatory milieu led to an altered tissue reconstitution after wound infliction. Thus, our data provide evidence that CD83 acts as a gatekeeper for the phenotype and function of pro-resolving Mφ.

## Introduction

Macrophages (Mφ) constitute not only a vital part of the first defense line against invading pathogens, but they also resolve ongoing inflammation to re-establish tissue homeostasis. This variety of tasks requires a high level of phenotypic and functional plasticity to adapt to diverse environmental cues (1, 2). For example, Mφ undergo specific phenotypic and functional changes thereby contributing to proper wound healing upon skin injury. The wound healing process is generally characterized by overlapping phases, i.e. hemostasis, formation of inflammatory tissue, proliferation and remodeling of injured tissue (3, 4). Mφ are central for wound closure during all these stages, which is reflected by aberrant wound healing processes when Mφ have been depleted (4). Mφ not only dispose cellular debris during the inflammatory stage of wound healing, but later they adopt to a pro-resolving phenotype and secrete trophic factors, such as FGF or TGF-β, that induce proliferation of fibroblasts to promote complete wound closure (5).

These multifaceted Mφ phenotypes, which are required to adapt to *in vivo* challenges such as wound healing, are often classified into a spectrum between the two polar extremes of IFN-γ-stimulated, classically activated Mφ (CAM), and IL-4-treated, alternatively activated Mφ (AAM) (6). Pro-inflammatory CAM (MHCII^high^, CD86^high^, MerTK, CD40) predominantly boost inflammation by secretion of pro-inflammatory cytokines/chemokines, such as TNF-α, IL-6, IL-1β, CCL2, RANTES (7, 8). By contrast, alternatively activated Mφ (AAM) are often referred to as pro-resolving Mφ (MHC-II^low^, CD86^low^, CD206, PDL2, CD200R, MSR-1), that express specific mediators, such as CCL22, CCL17 or IL-10 (9, 10). Although the dichotomy of CAM and AAM does not suffice to grasp the entire complexity of Mφ polarization *in vivo*, it can be a valuable substitute for studying several aspects of Mφ biology *in vitro* (11). In this regard, genes that are associated uniquely with one of these phenotypes might emerge as important regulators of Mφ function.

Microarray analyses revealed that the *Cd83* transcript is specifically induced in AAM but not CAM (7). The corresponding membrane bound CD83 (mCD83) glycoprotein, which is expressed on activated immune cells, has been described to have potent immunomodulatory properties (12, 13). Furthermore, CD83 inhibits the ubiquitin-dependent degradation of MHC-II and CD86 on DCs as well as MHC-II on thymic epithelial cells, mediated by MARCH1 and MARCH8 respectively, thereby stabilizing the surface expression of these important molecules (14, 15). Moreover, CD83 expressed by DCs and regulatory T cells (Tregs) plays a central role in promoting resolution of inflammation (16, 17). In addition, a soluble isoform of CD83 (sCD83) has also been described, having profound immunomodulatory properties in murine autoimmune and transplantation models (12, 13, 18, 19). Recently, we reported that sCD83 induces pro-resolving Mφ, thereby improving corneal transplant survival (20) as well as skin wound healing processes (21).

However, the role of endogenously expressed mCD83 by Mφ is less well understood. An early study revealed that CD83 is preformed and stored intracellularly in human monocyte-derived cells, which allows for rapid surface display after stimulation with LPS. While LPS-stimulated DCs stably express CD83 for up to 48 hours, CD83 is only transiently detectable on monocytes and Mφ upon LPS stimulation, suggesting distinct regulatory mechanisms that might also affect cellular functions (22). Even more importantly, the role of CD83 expressed by AAM has not yet been addressed, despite the clear association with this Mφ phenotype (7).

Thus, we first analyzed CD83 expression kinetics in murine bone-marrow derived Mφ (BMDM) after stimulation with pro- and anti-inflammatory agents. Like in human Mφ, CD83 is expressed only transiently by BMDM after LPS stimulation, but shows stable surface display after stimulation with IL-4, suggesting an association with a pro-resolving phenotype. To further investigate the biological function of CD83 expressed by Mφ, we generated conditional knock-out (cKO) mice, specifically lacking CD83 expression by Mφ (CD83^ΔMφ^). BMDM from cKO mice show a striking reduction of MHC-II and CD86 expression, which could be explained by the missing inhibition of MARCH1, mediated by mCD83 (14). Stimulation of CD83-deficient Mφ with IL-4 resulted in a disturbed homeostatic IL-4 phenotype, characterized by lower expression levels of the inhibitory CD200R and scavenger receptor MSR-1, whilst DECTIN-1 was upregulated. We report data suggesting that this phenotype is associated with decreased IL-4 signaling activity. Functionally, CD83-deficient Mφ are characterized by their impaired phagoytic activity, by a pro-inflammatory cytokine signature as well as an enhanced allogeneic T cell stimulatory capacity. Using a full-thickness excisional wound healing model, we show that the specific deletion of CD83 in murine Mφ boosts the inflammatory phase within the wound area. This was hallmarked by an accelerated wound closure on day 3 in cKO mice compared to CD83wt control mice. As mentioned above during later phases of normal wound healing, i.e. without scar formation or fibrosis, Mφ adapt to an anti-inflammatory phenotype. However, we did not observe these phenotypic changes, since our analyses revealed increased expression of pro-inflammtory CAM-associated transcripts, e.g. *Il6* and *Cxcl1* as well as an decreased expression of AAM-associated transcripts including *Cd200r, Msr-1* as well as *Ym-1.* Thus, these data confirm our *in vitro* analyses showing an increased inflammatory phenotype of CD83-deficient macrophages. Finally, this pro-inflammatory milieu in wound areas of CD83-deficient cKO mice resulted in the upregulation of fibrosis associated transcripts, such as *Tgfb, Acta-2* and *Col1a1* on day 6.

Collectively, here we report for the first time data regarding the regulation of CD83 expression by murine Mφ and characterize CD83 as a checkpoint molecule modulating the function of murine Mφ.

## Results

### Stimulation of the IL-4 signaling pathway results in long-term and stable expression of CD83 on Mφ

**Figure 1:**
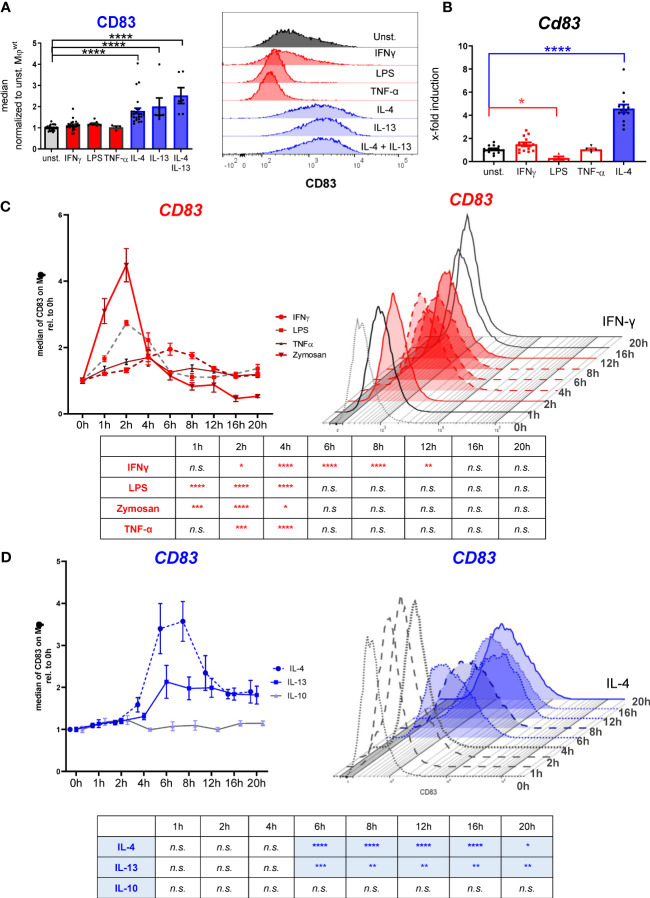
Analyses of CD83 surface expression by murine bone-marrow derived Mφ using different stimuli. Murine bone-marrow derived macrophages were generated and CD83 expression levels were analyzed after inflammatory or alternative activation. **(A)** Flow cytometric analyses show no CD83 expression on murine Mφ upon stimulation with IFN-ɣ (300 U/ml), LPS (100ng/ml), TNF-α (300 U/ml) for 16h. In contrast, stimulation with IL-4 (40 ng/ml), IL-13 (40 ng/ml) or IL4+IL-13 resulted in high CD83 expression on the surface of Mφ at the 16h time point (n ≥ 4) (left bar graph); Representative FACS histograms are presented (right graph). **(B)** qPCR analyses of *Cd83* mRNA expression after different stimulations in murine Mφ. **(C)** Time-dependent regulation of CD83 expression on BMDM after inflammatory activation with IFN-ɣ (300 U/ml), LPS (100ng/ml), TNF-α (1000 U/ml) or Zymosan 10 µg/ml (n ≥ 4), analyzed by flow cytometry. **(D)** Time-dependent regulation of CD83 expression on BMDM upon stimulation with IL-4 (40 ng/ml), IL-13 (40 ng/ml) or IL-10 (10 ng/ml) (n ≥ 4), analyzed by flow cytometry. Gating strategy for the Mφ population is depicted in **Supplementary Figure 2**. Data are represented as mean ± SEM. Statistical analysis was performed using a Two-way ANOVA or the appropriate corresponding non-parametric test. Experiments were performed at least three times. n.s., not significant, which indicates there is no statistical signficance; * p<0.05; ** < 0.01; *** p < 0.001; **** p < 0.0001.

Previous studies reported that CD83 is transiently expressed on human Mφ after LPS stimulation and that it is associated with IL-4 induced gene expression in murine Mφ (7, 22). However, temporal regulation of CD83 expression after both pro- and anti-inflammatory stimulation of murine Mφ has not yet been investigated. Thus, we incubated murine BMDM either with pro-inflammatory or with anti-inflammatory mediators and assessed CD83 expression kinetics. Stimulation with IL-4 induced a strong and long-lasting CD83 expression on the cell surface of murine Mφ after 16 h, while neither LPS, IFN-γ nor TNF-α induced elevated surface expression levels (**Figure 1A**, left bar graph). Representative FACS histograms are shown in **Figure 1A**, right graph. In agreement with these results, we detected no *Cd83* regulation on mRNA level upon stimulation with IFN-γ or TNF-α after 16h (**Figure 1B**). In addition, LPS stimulation of murine Mφ results in significantly reduced *Cd83* mRNA levels in comparison to unstimulated Mφ (**Figure 1B**). Again, only IL-4 induced significant *Cd83* mRNA transcript levels after 16 h post stimulation (**Figure 1B**).

Subsequently, we investigated the temporal regulation of CD83 expression. Thus, we stimulated Mφ with LPS and other pro-inflammatory compounds and analyzed surface expression of CD83 over a 20 h time course (**Figure 1C**). LPS treatment caused an almost threefold increase of CD83 surface expression at the 2 h time point, followed by a rapid decline to baseline levels after six to eight hours. Similarly, the yeast cell wall component zymosan, which in contrast to LPS acts *via* TLR2, induced fourfold higher CD83 surface levels during the first two hours of stimulation. A slightly slower and less pronounced response was observed upon treatment with TNF-α, which resulted in a peak of CD83 expression after 4 h (**Figure 1C**). IFN-γ induced a rather delayed type of response, with a steady increase up to two-fold within the first six hours and a subsequent decrease until 16 h after stimulation (**Figure 1C**). Interestingly, IL-4 and IL-13 treatment caused a 2- to 3-fold CD83 induction as early as 4 h after stimulation, but in contrast to the pro-inflammatory mediators, this elevated expression did not revert to baseline levels even 20 h after stimulation (**Figure 1D**). Treatment with IL-10, another anti-inflammatory cytokine, had no influence on CD83 expression (**Figure 1D**). Thus, we observed a striking discrepancy in CD83 regulation after stimulation: while pro-inflammatory mediators induced a very rapid but transient increase in CD83 surface expression, stimulation of the IL-4R with either IL-4 or IL-13 resulted in a stable CD83 display on the cell surface. These data indicate an interesting functional role of CD83 in Mφ biology, especially for the resolving phenotype associated with IL-4 signaling.

### Cell specific deletion of CD83 expression in Mφ interferes with their pro-resolving phenotype

**Figure 2:**
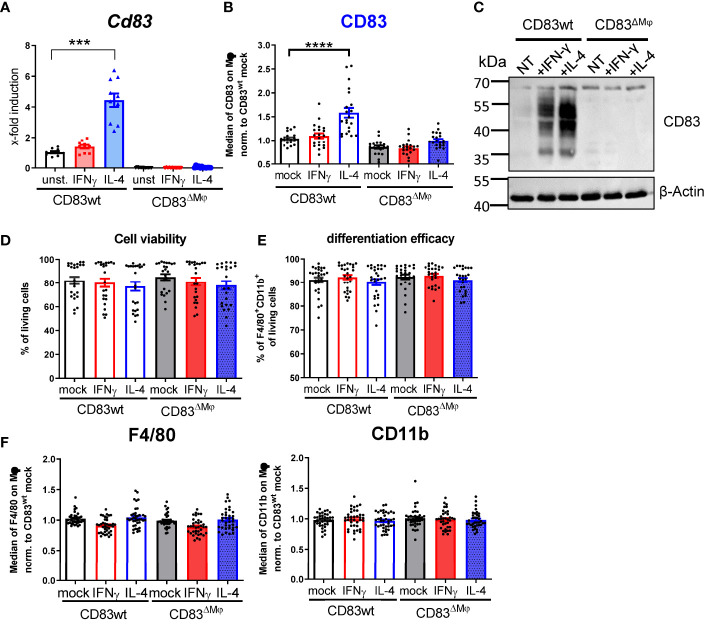
Analyses of CD83 deficient murine Mφ. Mφ were generated from CD83wt or CD83 cKO mice and subsequently stimulated with IFN-γ or IL-4 for 16h or left untreated. **(A)** *Cd83* expression levels were determined by qPCR and normalized to CD83wt BMDMs (n = 10). **(B)** Assessment of CD83 expression levels by flow cytometry (n = 20). **(C)** Assessment of knock-out efficiency in whole cell lysates from mock-, IFN-γ or IL-4 stimulated Mφ by Western blotting. β-actin served as a loading control. See full uncut gels in **Supplemental Material (S1)** **(D)** Cell viability assessment using flow cytometry (n = 24). **(E)** Differentiation efficacy assessing the percentage of F4/80^+^CD11b^+^ cells by flow cytometry, representing the Mφ population (n ≥ 24). **(F)** Expression levels of F4/80 and CD11b within the Mφ population (n ≥ 40). The gating strategy for the Mφ population is depicted in **Supplementary Figure 2**. Statistical analyses were performed by One-way ANOVA or the appropriate corresponding non-parametric test. Data are represented as mean ± SEM. Experiments were performed at least three times. ***p<0.001; **** p< 0.0001. The absence of asterisks indicates that there is no statistical significance.

Next, we aimed to characterize the biological function of CD83 expressed by murine Mφ by using a conditional knock-out (cKO) strategy. By crossing mice carrying floxed *Cd83* alleles with a CX3CR1-Cre line, we generated a conditional line with an abrogated CD83 expression specifically in Mφ (herein termed CD83^ΔMφ^). To test the efficacy of our KO strategy, we treated Mφ from CD83 cKO mice and wt mice with IFN-γ or IL-4 for 16 h and assessed the expression of *Cd83* by qPCR (**Figure 2A**). As depicted in **Figure 2A**, murine CD83wt Mφ stimulated with IL-4 show a highly significant induction of *Cd83* mRNA levels compared to unstimulated CD83wt Mφ, whilst no expression was observed in Mφ from cKO mice (**Figure 2A**). These results were confirmed by surface expression analyses of cKO derived Mφ after IL-4 treatment, whereas wt derived Mφ showed a significant and stable expression of CD83 (**Figure 2B**). Similarly, CD83 protein was also absent in cell lysates derived from cKO BMDMs as shown by Western blot analyses (**Figure 2C**). Interestingly, while stimulation with IFN-γ had no apparent effect on CD83 surface expression after 16 h, total protein levels were also elevated (**Figure 2C**), although to a lesser extent than IL-4 treatment. Next, we examined whether CD83-deletion affects cell viability (**Figure 2D**) or differentiation efficacy (**Figure 2E**), by flow cytometry and found that neither were affected. Furthermore, CD83 deletion has no effect on expression levels of F4/80 as well as CD11b on CD83^ΔMφ^ (**Figure 2F**). Thus, we conclude that CD83 deletion does not alter cell viability nor differentiation of murine mock-, IFN-γ- or IL-4-stimulated BMDM, generated from CD83wt or cKO mice.

**Figure 3:**
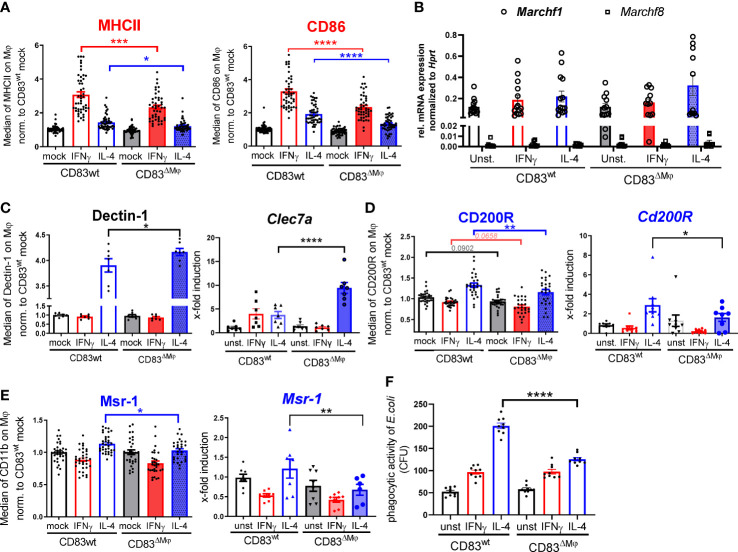
CD83 deficiency modulates the pro-resolving phenotype of IL-4 stimulated Mφ. BMDM were generated and differentiated into inflammatory CAM or AAM, using IFN-γ or IL-4 respectively, for 16h. **(A)** Assessment of surface MHC-II (n ≥ 47) and CD86 (n ≥ 47) expression levels by flow cytometry on stimulated murine wt and CD83-deficient Mφ. **(B)** qPCR analyses of *Marchf1* and *Marchf8* in Mφ derived from CD83wt or CD83 cKO mice (n = 5). **(C)** Analyses of Dectin-1 surface expression levels (left bar graph) and mRNA expression levels (right bar graph, *via* flow cytometry and qPCR, respectively). Significantly increased Dectin-1 expression levels on IL-4 stimulated CD83-deficient Mφ (n = 7). **(D)** Analyses of CD200R surface expression (left bar graph, n ≥ 29-31) and mRNA expression (right bar graph, n ≥ 8) revealed significantly decreased levels on IL-4 stimulated CD83-deficient Mφ **(E)** Analyses of MSR-1 surface expression (left bar graph, n ≥ 31) and Msr-1 mRNA expression (right bar graph, n = 6) revealed significantly decreased levels on IL-4 stimulated CD83-deficient Mφ. **(F)** Assessment of phagocytic activity *via* gentamicin protection assays revealed significantly decreased capacity to engulf *E.coli*. Statistical analyses were performed by One-way ANOVA or the appropriate corresponding non-parametric test. Data are represented as mean ± SEM. Experiments were performed at least three times. *p< 0.05; **p<0.01; ***p<0.001; **** p< 0.0001. The absence of asterisks indicates that there is no statistical significance.

In APCs, such as DCs and B cells, CD83 has been reported to stabilize surface MHC-II and CD86 expression by preventing their ubiquitin-dependent degradation (14, 17). Both molecules are hallmarks of a classic activation *via* IFN-γ, and consequently, we addressed the question how deletion of CD83 might affect their surface display. We observed an up-regulation of MHC-II and CD86 molecules on BMDM after stimulation with IFN-γ and to a lesser extent after IL-4 treatment (**Figure 3A**). In line with previous reports regarding the CD83-MARCH-MHC-II axis, Mφ from CD83^ΔMφ^ mice exhibited significantly lower surface expression levels of MHC-II and CD86 (**Figure 3A**). Since CD83 inhibits MHC-II ubiquitination *via* the interference with the ubiquitin-ligase March1 or March8 in DCs or thymic epithelial cells, respectively (14, 15), we tested which ubiquitin-ligase predominates in BMDM. Thus, BMDM were either left unstimulated or treated with IFN-γ, and we detected comparable levels of *Marchf1* transcripts. Since *Marchf8* expression is reduced by almost two orders of magnitude in comparison to *Marchf1* (see. **Figure 3B**), we conclude that CD86 as well as MHC-II stabilization is achieved by CD83-mediated inhibition of March1.

Next, we analyzed the overall phenotype of CD83-deficient Mφ. Since CD83 expression is tightly associated with an IL-4 mediated alternative activation (**Figure 1C**), we hypothesized that deletion of CD83 would mostly affect polarization of AAM. Indeed, we detected an altered phenotype in IL-4 stimulated CD83-deficient Mφ. This phenotype was characterized by significantly increased Dectin-1 expression on protein (**Figure 3C**, left bar graph) and mRNA level (**Figure 3C**, right bar graph), which is associated with a pro-inflammatory CAM polarization (23–26). Concomitantly, in IL-4-stimulated CD83-deficient Mφ we observed significantly reduced expression levels of the inhibitory receptor CD200R, which is known to limit pro-inflammatory cytokine secretion (27) (**Figure 3D**, left bar graph**)**. Moreover, pro-resolving MSR-1 was also significantly decreased in IL-4 stimulated CD83-deficient Mφ, compared to CD83wt control Mφ. Furthermore, qPCR analyses of *Clec7a* (**Figure 3C**, right bar graph), *Cd200R* (**Figure 3D**, right bar graph) and *Msr-1* (**Figure 3E**, right bar graph) are in line with the protein data. Msr-1 is known to be upregulated on AAM being important to phagocytose *E.coli* bacteria. Since we observed a reduction in Msr-1 expression, we next checked whether CD83-deficient Mφ were impaired in their phagocytic activity to engulf *E.coli.* In fact, using a gentamicin protection assay revealed a significantly impaired phagocytic activity of IL-4 stimulated, CD83-deficient Mφ (**Figure 3F**). Collectively, these data suggest a profound functional change of IL-4 stimulated, CD83-deficient Mφ.

### CD83-deficient Mφ show a reduced phosphorylation status of STAT-6 upon IL-4 stimulation

**Figure 4:**
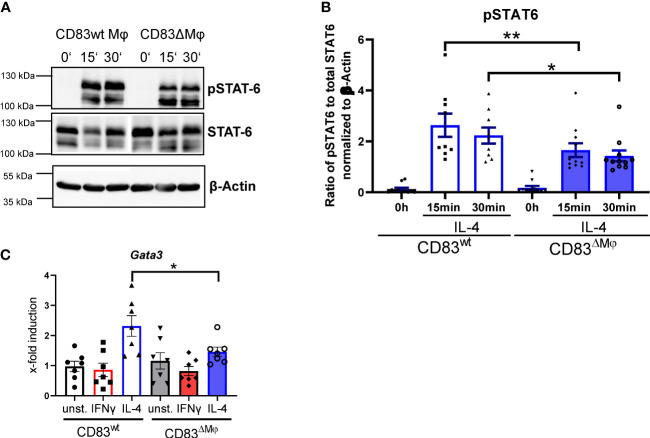
STAT6 phosphorylation is altered in CD83-deficient macrophages upon IL-4 stimulation. Bone-marrow derived Mφ were generated from CD83wt or CD83 cKO mice and stimulated with IL-4 for 15 or 30 min. Unstimulated Mφ served as control. Subsequently, whole cell lysates were prepared and analyzed by Western blot. **(A)** Representative Western blot showing pSTAT-6, STAT6 and β Actin levels in whole cell lysates derived from CD83wt and CD83ΔMφ animals **(B)** Quantification of the ratio of pSTAT-6 and STAT6 normalized to β-actin (n ≥ 9). **(C)** qPCR analyses of *Gata3* mRNA levels in Mφ generated from cKO or wt mice, stimulated with IFNg or IL-4. Significantly reduced *Gata3* mRNA levels are observed in IL-4 stimulated CD83-deficient Mφ. Statistical analyses were performed by One-way ANOVA or the appropriate corresponding non-parametric test. Data are represented as mean ± SEM. Experiments were performed at least three times. *p< 0.05; **p<0.01. The absence of asterisks indicates that there is no statistical significance.

Since we detected a phenotypic change on IL-4-stimulated Mφ derived from CD83 cKO mice, we next examined whether members of the IL-4 signaling cascade are also modulated. IL-4 binds to IL-4Rα that recruits the IL-2Rγ chain, which leads to the activation of the tyrosine kinases Jak1/Jak3 and phosphorylation of STAT6, which form pSTAT6-dimers and translocate to the nucleus and initiate transcription of target genes (28, 29). To analyze possible differences between CD83wt and CD83 KO Mφ, we generated Mφ from CD83wt as well as CD83 cKO mice and stimulated them with IL-4 for 15 or 30 min. Subsequently, whole-cell lysates were prepared and analyzed by Western blot, in respect to pSTAT6 and STAT6 levels. In fact, we detected a decreased phosphorylation status of STAT6 upon IL-4 stimulation in CD83-deficient Mφ, compared to CD83wt Mφ (**Figure 4A**). Quantified ratios of pSTAT6 to STAT6 are shown in **Figure 4B**. Next, we analyzed the expression of STAT6 target gene *Gata3* (30), and its expression was downregulated in IL-4-stimulated CD83-deficient Mφ, when compared to wt derived Mφ (**Figure 4C**).

### CD83-deficient Mφ show increased pro-inflammatory cytokine production and enhanced T cell stimulatory capacity

**Figure 5:**
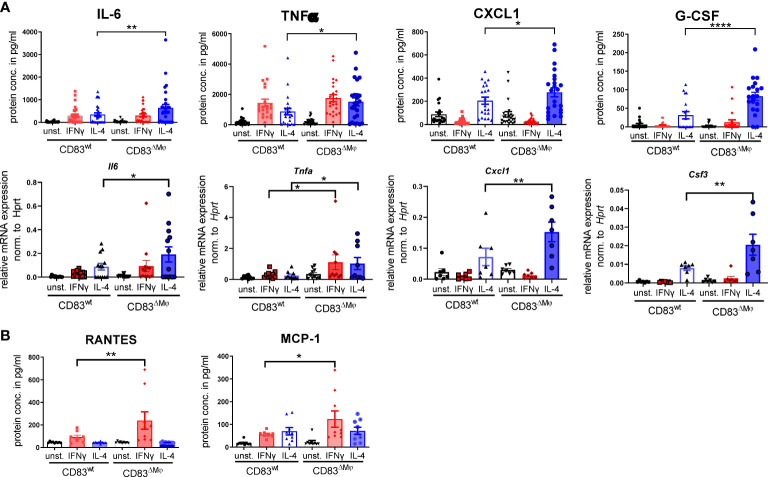
CD83-deficient IL-4- as well as IFN-γ-stimulated Mφ show a pro-inflammatory profile. Bone-marrow derived Mφ were generated and differentiated either into CAM or AAM, *via* IFN-γ or IL-4 respectively, or were left untreated for 16h. Afterwards, the supernatants were analyzed by CBA and cells *via* qPCR. **(A)** IL-4 stimulated CD83-deficient Mφ show increased secretion levels of IL-6, TNF-α, CXCL1 and G-CSF (upper bar graphs). qPCR analyses showed significantly increased mRNA levels of *Il-6, Tnfa, Cxcl1 and Csf3* in IL-4-stimulated CD83 KO Mφ (lower bar graphs). **(B)** CCL5/RANTES and MCP-1 expression levels are increased in supernatants of IFN-γ-stimulated CD83-deficient Mφ. Statistical analyses were performed by One-way ANOVA or the appropriate corresponding non-parametric test. Data are represented as mean ± SEM. Experiments were performed at least three times. *p< 0.05; **p<0.01; **** p< 0.0001. The absence of asterisks indicates that there is no statistical significance.

**Figure 6:**
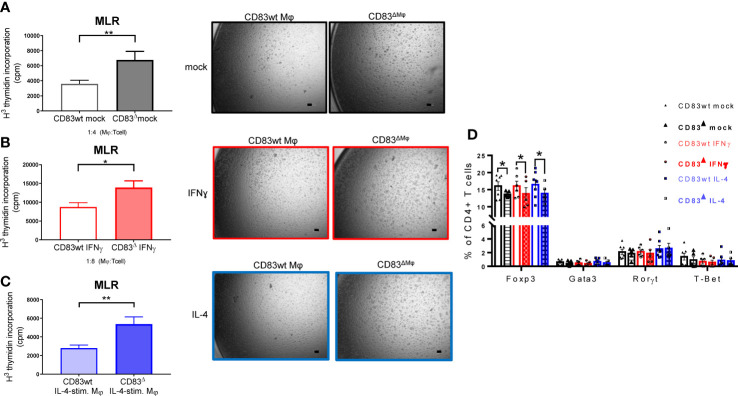
CD83-deficient Mφ show enhanced capacity to stimulate allo-reactive T cells. Mφ were generated from CD83wt and CD83 cKO mice and differentiated using IFNγ or IL-4. Afterwards, the medium was discarded and splenocytes derived from BALB/c mice (4x10^5^ cells/well) were co-cultured with differentiated Mφ in 96-well plates, at different Mφ:splenocyte ratios, as indicated for 48h. T cell proliferation was assessed using tritium **(A–C)**. Co-cultures of unstimulated, IFN-γ- and IL-4 stimulated Mφ, derived from CD83-deficient Mφ, show enhanced proliferative responses, when compared to co-cultures with CD83wt derived Mφ (left bar graphs, **A–C**). This observation is reflected by decreased T cell clusters shown in representative microscopic images (**A–C**, right side). **(D)** Flow cytometric analyses revealed a significantly decreased frequencies of Tregs (CD4^+^Foxp3^+^ cells) in co-cultures of CD83-deficient Mφ with allo-reactive splenocytes. Statistical analyses were performed by using an Unpaired t-test **(A–C)** or Two-way ANOVA **(D)** or the appropriate corresponding non-parametric test (n ≥ 4). Data are represented as mean ± SEM. Experiments were performed at least three times. *p< 0.05; **p<0.01. The absence of asterisks indicates that there is no statistical significance.

Next, we analyzed the impact of CD83-deficiency regarding the cytokine and chemokine production of IL-4- and IFN-γ-stimulated Mφ. As depicted in **Figure 5A**, IL-4-stimulated CD83-deficient Mφ showed a significantly increased secretion of pro-inflammatory mediators, including IL-6, TNF-α, CXCL1, and G-CSF (**Figure 5A** upper bar graphs). Additionally, we verified these data by qPCR and observed significantly increased expression levels of the corresponding transcripts *Il6*, *Tnfa*, *Cxcl1*, and *Csf3* (**Figure 5A** lower bar graphs). In addition, we detected significantly increased levels of RANTES/CCL5 (**Figure 5**, left bar graph) and of MCP-1/CCL2 in IFN-γ-stimulated CD83-deficient Mφ (**Figure 5**, right bar graph), suggesting that CD83 also influences the function of IFN-γ stimulated Mφ. Collectively, these data support our hypothesis that CD83 deficiency modulates activation and function of Mφ. Recently we reported that CD83-deficient DCs enhance antigen-specific T cell proliferation and increase secretion of pro-inflammatory cytokines compared to co-cultures with CD83wt DCs (17). Since the data described above indicate a modulated pro-resolving phenotype of CD83-deficient Mφ, we next assessed the T cell stimulatory capacity of IFN-γ- and IL-4-stimulated, in comparison to unstimulated Mφ, generated from CD83wt and CD83 cKO mice. Thus, we co-cultured the differently stimulated Mφ with allogeneic splenocytes, derived from BALB/c mice, and T cell proliferation was assessed *via* tritium incorporation. As depicted in **Figure 6**, T cell proliferation was enhanced in all co-cultures with CD83-deficient Mφ, regardless of their stimulus (**Figures 6A–C**). This observation is also reflected by enhanced clustering of T cells upon co-culture with CD83-deficient BMDMs (see representative microscopic images¸ **Figures 6A–C**). In MLRs with CD83-deficient Mφ we observed significantly increased proliferative response of alloreactive T cells (**Figures 6A–C**). Next, we investigated if the composition of T cell subsets, present in co-cultures with mock-, IFN-γ or IL-4-treated Mφ generated from CD83wt or CD83 cKO mice, would be altered. As depicted in **Figure 6D**, flow cytometric analyses revealed significantly reduced frequencies of CD4^+^Foxp3^+^ Tregs in co-cultures of allogeneic splenocytes with mock, IFN-γ or IL-4-stimulated CD83-deficient Mφ. This indicates that the reduced numbers of Tregs present in the co-cultures may account for the observed increased T cell proliferation (**Figures 6A–C**).

### CD83-deficient Mφ accelerate the inflammatory phase of wound healing and promote upregulation of fibrosis associated transcripts

**Figure 7:**
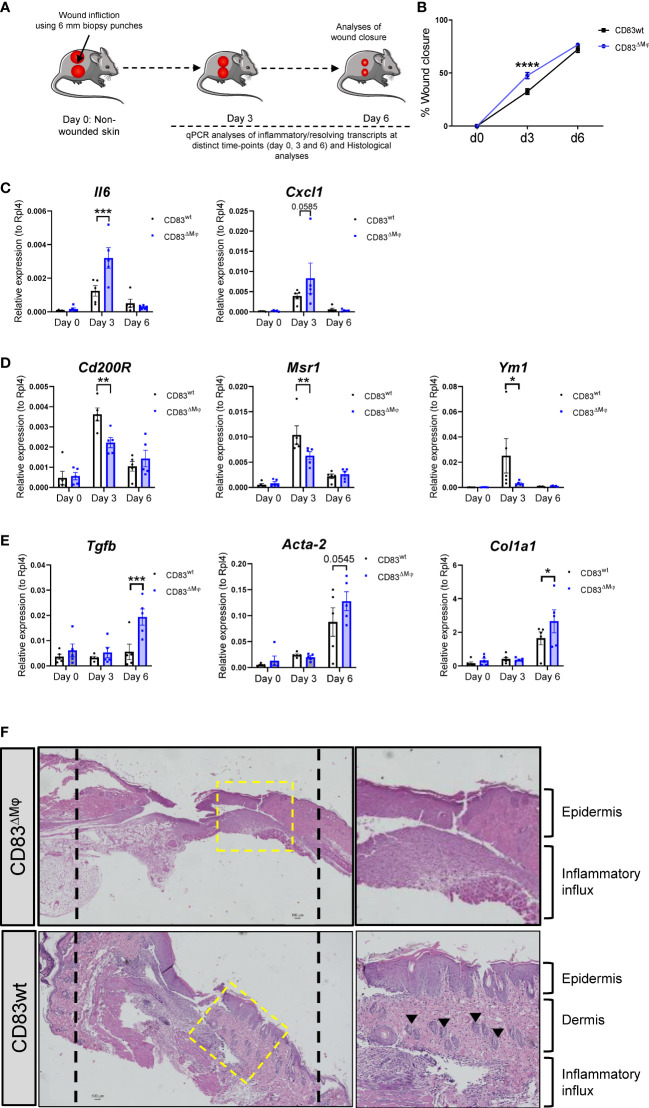
CD83-deficient Mφ accelerate the inflammatory phase of wound healing and promote upregulation of fibrosis associated transcripts **(A)** Experimental set-up for the full-thickness excisional wound healing model. Biopsy punches (6mm) were placed into the dorsal skin of CD83wt as well as CD83 cKO mice. **(B)** Wound closure was calculated relative to the initial d0 wound dimension. 8mm silicone rings (Thermo scientific) were mounted around the wound area, using vetbond (3M). Imaging was performed on day 0, 3, and 6 and wound diameters were determined by ImageJ. **(C–E)** qPCR analyses were performed using skin biopsies from day 0, 3 and 6 (n = 5 per group). **(F)** Representative H&E slides of day 6 wound biopsies from CD83wt as well as CD83 cKO mice. Statistical analyses were performed by using a Two-way ANOVA or the appropriate corresponding non-parametric test. Data are represented as mean ± SEM. *p< 0.05; **p<0.01; ***p<0.001; **** p< 0.0001. The absence of asterisks indicates that there is no statistical significance.

In order to investigate the *in vivo* relevance of CD83-deficiency in CX3CR1^+^ Mφ, we performed full-thickness excisional wound healing experiments using cKO (CD83^ΔMφ^) in comparison to wildtype control mice (CD83wt). As depicted in **Figure 7A**, we induced 6 mm biopsy punches in the dorsal skin and monitored wound closure until day 6 and collected skin biopsies on day 3 as well as day 6 after wound infliction. As shown in **Figure 7B**, on day 3 the wound closure was significantly enhanced in CD83^ΔMφ^ mice when compared to CD83wt mice. This indicates a boost of the initial inflammatory phase, which is crucial for wound closure. This upregulated inflammatory phase was also confirmed by qPCR analyses, since transcripts such as *Il6* and *Cxcl1*, associated with a pro-inflammatory macrophage phenotype, were upregulated in CD83^ΔMφ^ mice (**Figure 7C**). Concomitantly, markers associated with pro-resolving Mφ, including *CD200r, Msr-1* as well as *Ym-1* were significantly reduced (**Figure 7D**). Surprisingly, regarding surface wound closure on day 6, no differences were detected between CD83^ΔMφ^ and wt animals. However, in samples derived from cKO animals we observed significantly increased expression levels of *Tgfb*, *Acta-2* as well as *Col1a1.* This indicates a higher prevalence of myofibroblasts and fibrosis associated transcript in CD83^ΔMφ^ mice, which are associated with disturbed wound healing processes (**Figure 7E**). Indeed, histological analyses of day 6 skin biopsies showed an altered reconstitution process, as indicated by an expanded epidermis, absent dermis and a prominent inflamed tissue area in CD83^ΔMφ^ mice (**Figure 7F**, upper panel). In contrast, histological analyses of skin biopsies from CD83wt mice showed a distinct epidermis alongside the dermis and a resolving inflammatory area (**Figure 7F**, lower panel). Of note, in CD83wt mice, hair follicles have already migrated into the former sites of excisional wounds, indicating a progressed stage of wound regeneration, which was not observed in CD83^ΔMφ^ mice.

## Discussion

Mφ are cells which show highly phenotypic plasticity in response to environmental cues. Despite a multitude of functionally diverse activation states observed *in vivo*, Mφ are often classified into two distinct polar extremes, namely IFN-γ-stimulated CAM with pro-inflammatory capacities and anti-inflammatory IL-4-treated AAM. CAM and AAM reveal striking differences in their transcriptome and subsequently provide specifically tailored effector functions during immune responses (8, 31). CAMs are essential during the initial inflammatory phase of wound healing. However, if they are still present during later stages, without switching to a pro-resolving, tissue repair AAM-associated phenotype, they are rather associated with incomplete/poor wound healing and fibrosis (32, 33). In contrast, anti-inflammatory AAMs are pivotal for resolution of inflammation and tissue regeneration during the later stage of wound healing (34, 35).

The crucial involvement of Mφ in numerous major health-threats, such as chronic inflammatory/autoimmune diseases and cancer, render these cells ideal targets for immunomodulatory interventions. Identification of novel immune checkpoint molecules on Mφ that stabilize either an inflammatory or pro-resolving phenotype and function can lead to the development of new therapeutic agents for the treatment of the respective disease.

One promising candidate is the CD83 protein, which has been described as an important checkpoint molecule that favors resolution of inflammation. In the context of autoimmune diseases and transplantation, several studies demonstrated that the sCD83 protein promotes resolution of inflammation and induces tissue tolerance (13, 18–20, 36–38). Studies using conditional KO (cKO) mice showed that deletion of CD83 in Tregs results in a pro-inflammatory Treg phenotype, which was characterized by increased levels of TNF-α and IL-1β concomitantly impaired secretion of pro-resolving molecules e.g. IL-10 and TGF-β (16). Analogously, conditional knockout mice with specific CD83 deletion in DCs showed excessive inflammatory autoimmune responses and impaired resolution of inflammation (17, 39). However, little was known regarding the regulation and function of CD83 expressed by Mφ.

An early study compared the expression pattern of CD83 upon LPS stimulation in human monocytes/macrophages as well as human DCs and showed that CD83 is highly and stably expressed on mDCs but not on Mφ (22). Furthermore, microarray analyses revealed that Mφ treated with IL-4, but not with IFN-γ+LPS, express *Cd83*, alongside typical AAM-associated gene transcripts, such as *Fizz1* and *Arg1* (7). This associates CD83 with the pro-resolving AAM phenotype, which is linked to tissue-repair and resolution of inflammation.

In order to investigate whether CD83-deletion interferes with the CAM or AAM phenotype, we subsequently performed different *in vitro* assays. Phenotypic analyses of CD83 cKO Mφ reinforced the concept of a tight connection between CD83 expression and an AAM-phenotype. In line with previous literature, CD83-deficient Mφ display decreased expression levels of the costimulatory molecule CD86 as well as MHC-II (**Figure 3**), which is most likely due to the missing blockade of the ubiquitin-ligase MARCH-1 in CD83 deficient Mφ (14). We attribute this effect to MARCH-1, as it is the prevailing MARCH-ubiquitinase expressed by Mφ, while MARCH-8, which is blocked by CD83 in thymic epithelial cells (15), is only marginally expressed. Furthermore, in IL-4 stimulated CD83-deficient Mφ we detected a striking reduction of the AAM-associated molecule CD200R, which is crucial to control inflammatory responses by limiting pro-inflammatory cytokine secretion and cellular function (27, 40). This further supported the notion of a disturbed pro-resolving AAM-phenotype upon CD83 deletion. Scavenger receptors such as MSR-1 are involved in phagocytosis of cellular debris, which is a hallmark of AAM and crucial for proper resolution of inflammation (41, 42). Consequently, the reduction of MSR1 resulted in an impaired phagocytic activity upon IL-4 stimulation and additionally, CD83-deficient Mφ show a pronounced pro-inflammatory cytokine profile. Thus, we conclude that CD83 expressed by Mφ is crucial for the resolving AAM phenotype and function, which is crucial for resolution of inflammation.

The modified alternative activation state of CD83 cKO Mφ towards pro-inflammatory features is further underpinned by the reduced phosphorylation status of STAT6, which is essential for the induction of AAM-related transcripts (43). In line with this knowledge, we found decreased expression levels of the STAT6 target gene *Gata3*, which is also a prominent marker of IL-4 stimulated AAM (30). Since STAT6 is responsible for the anti-inflammatory properties of murine Mφ (44), we conclude that the perturbed phenotype of IL-4 stimulated CD83-deficient Mφ is linked to the decreased STAT6 signaling activity. The notion that CD83 cKO derived Mφ are further modulated towards a defect in resolving functions is further underlined by the fact that we detected elevated expression levels of Dectin-1 after IL-4 treatment. Although induced by IL-4, expression of Dectin-1 is rather linked to a pro-inflammatory CAM phenotype (26, 45). This is in line with the observed enhanced production of pro-inflammatory cytokines as well as chemokines by CD83 cKO derived Mφ.

Interestingly, CD83 cKO BMDM induced higher proliferative responses upon co-culture with allogeneic T cells, regardless of the preceding stimulation. These results parallel those obtained from CD83-deficient DCs, also showing an over-activated phenotype characterized by an upregulation of co-stimulatory molecules and pro-inflammatory cytokines, resulting in an enhanced antigen-specific T cell stimulation (17). In the present study, we extend these findings to Mφ derived from CD83 cKO mice and reveal that CD83 deficiency affects regulatory T cell numbers in allogeneic co-cultures. This again substantiates the fact that membrane-bound CD83 expression by Mφ is an important checkpoint molecule that contributes to resolution of inflammation by Treg induction. In a recent study, we have shown that administration of the soluble CD83 molecule during differentiation of murine Mφ results in a modulation towards an anti-inflammatory phenotype, which is able to induce tissue tolerance in a corneal transplantation model *in vivo*, which goes along with a decreased capacity to stimulate allogeneic T cells (13). Moreover, we have shown that sCD83 modulates Mφ towards a pro-resolving, tissue-repair AAM-associated phenotype, able to restore tissue function and proper wound healing (21). Within the present study, we confirm the pro-resolving function of membrane-bound CD83 expressed by murine Mφ, using an *in vivo* wound healing model. Although wound closure of cKO mice was significantly accelerated on day 3, which can be explained by an enhanced pro-inflammatory phenotype of CD83-deficient Mφ, later phases of tissue repair and resolution of inflammation were hampered. This is reflected by significantly increased expression levels of fibrosis associated transcripts, including *Acta-2* and *Tgf-b*, which have been linked to fibrotic scar formation (33, 46). In addition, our histological analyses revealed a disturbed wound healing process in CD83^ΔMφ^ mice.

**Figure 8:**
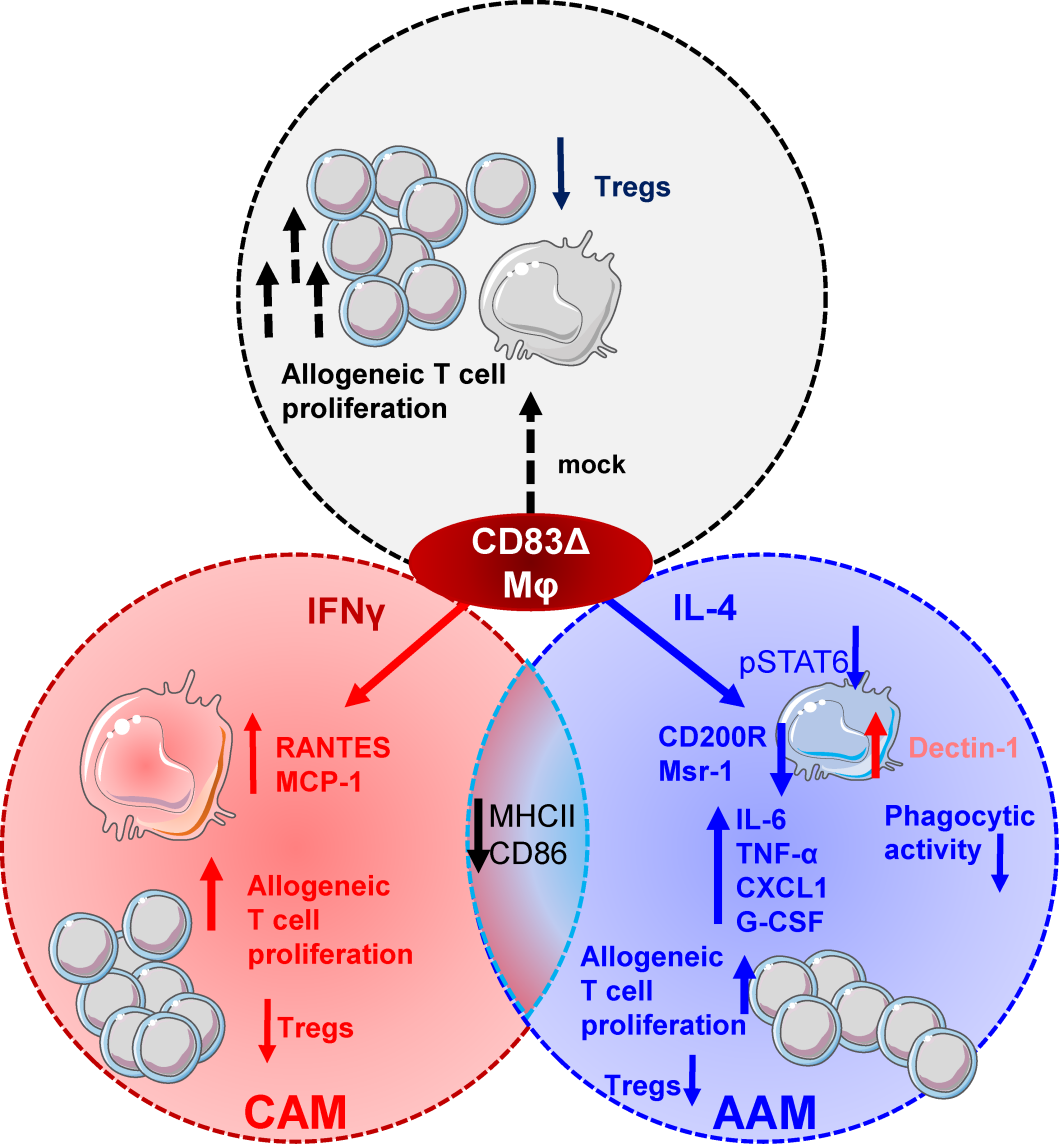
CD83 expressed by Mφ is an important immune checkpoint molecule that contributes to resolution of inflammation. CD83 is an early marker for IL-4 stimulated AAMs and its deletion in Mφ results in striking phenotypic and functional changes. CD83-deficient, IL-4 stimulated Mφ are characterized by a decreased STAT-6 phosphorylation status when compared to CD83wt Mφ. This goes along with reduced expression levels of AAM-associated marker molecules such as CD200R and Msr-1. Reduction in MSR-1 expression correlates with a reduced phagocytic activity of *E.coli* in CD83-deficient IL-4 stimulated Mφ. In contrast, CAM associated Dectin-1 expression is upregulated. Furthermore, CD83-deficient, IL-4 stimulated Mφ express increased levels of pro-inflammatory modulators, such as IL-6, TNF-α, CXCL1 and G-CSF. Functionally, Mφ generated from CD83 cKO mice show enhanced allogeneic T cell proliferative capacities and reduced frequencies of Tregs in Mφ - cell co-cultures. Finally, IFNγ-stimulated Mφ generated from cKO mice show an increased production of RANTES and MCP-1, indicating that CD83 also modifies the production of these pro-inflammatory chemokines.

Collectively, our data indicate that CD83 expression by Mφ is vital for the transition of pro-inflammatory Mφ into a pro-resolving, tissue-repair associated phenotype (see also **Figure 8**) and identifies CD83 as a potential target for future therapeutic intervention strategies.

## Materials and methods

### Mice

To generate mice with CD83-deficient Mφ, we used a conditional knock-out strategy (CD83 cKO) by mating mice with floxed *Cd83* alleles (47), with the *Cx3cr1*-Cre line, which was kindly provided by Prof. Dr. Gerhard Krönke (Department of Medicine 3, University Hospital Erlangen, Erlangen, Germany). Cre-negative littermates as well as age-matched Cre-positive CD83wt mice served as controls (hereafter referred to as wt mice). Animal care and all experimental procedures of the present study were performed in accordance with the European Community Standards on the Care and Use of Laboratory Animals and were approved by the local ethics committee.

### Generation and stimulation of bone-marrow-derived Mφ

Bone-marrow derived Mφ were generated from murine bone-marrow precursor cells from CD83 cKO mice and wild type littermates, in D10 medium consisting of DMEM (Lonza), 10% FCS (Merck), Penicillin-Streptomycin-Glutamine-solution (Sigma Aldrich) and 50 µM β-mercaptoethanol. Bone-marrow cells were flushed from femur and tibia of mice and seeded for 1d in D10 medium containing M-CSF supernatant (10 - 30%). After overnight incubation, cells were harvested and seeded at a starting density of 3-4 x 10^6^ cells per 10 cm^2^ dish (Falcon) in D10 medium + M-CSF supernatant. Fresh D10 medium + M-CSF was added on day 3. On day 6, Mφ were harvested and stimulated as described below.

### Stimulation of bone-marrow derived Mφ

On day 6, Mφ were harvested with 10 mM EDTA-PBS, washed with fresh medium and seeded in uncoated 24-well plates at a cell density of 2 x 10^6^ cells per ml. For phenotypic and functional characterization of CD83, Mφ were generated from wt or cKO mice and seeded for differentiation into classically activated Mφ (CAM) or alternatively activated Mφ (AAM) using IFN-γ (300 U/ml) or IL-4 (40 ng/ml, PeproTech), respectively. For time kinetic experiments, Mφ were stimulated with inflammatory activators such as IFNγ (300 U/ml, PeproTech), LPS (100 ng/ml, *In vivo*gen) or TNF-α (1000U/ml) or alternatively with mediators, such as IL-4 (40 ng/ml), IL-13 (40 ng/ml) or IL-10 (10 ng/ml) for the indicated time period. Subsequently, cells were analyzed by flow cytometry.

### Flow cytometric analyses

Live/dead discrimination was performed using either 7-AAD or LIVE/DEAD™ Fixable Aqua Dead Cell Stain (ThermoFisher Scientific). Surface staining of BMDMs and cells used in MLR assays was performed in PBS-diluted appropriate antibodies for 30 minutes. In the case of live/dead discrimination with 7-AAD, the dye was added just before the flow cytometry measurement. For intracellular staining, cells were permeabilized and fixed in Permeabilization Reagent (Thermo Fisher Scientific, 00-5523-00). The following antibodies were used from BioLegend, others are stated: F4/80 (BM8), CD11b (M1/70), CD200R (OX-110), Msr-1 (M204PA; Invitrogen), MHCII (M5/114.15.2), CD86 (GL-1), CD206 (C068C2), MERTK (2B10C42), CD83 (Michel-19) RORɣT (Q31-378), GATA3 (L50-823;BDBiosciences), T-BET (O4-46,BD Pharmingen), FOXP3 (FJK-16s; Thermo Fisher Scientific). Afterwards, the cells were washed with PBS and subsequently analyzed by flow cytometry.

### Cytometric bead array

Supernatants of BMDM were analyzed using the LEGENDplex™ Mouse Macrophage/Microglia or LEGENDplex™ Mu Pro-inflammatory Chemokine Panel (BioLegend), respectively, according to the manufacturer’s instructions.

### Mφ-allogeneic splenocyte cocultures (mixed lymphocyte reaction)

On day 6, BMDMs from wt controls and cKO mice were harvested, seeded in 96-well plates and stimulated with IFN-γ or IL-4 or left untreated. Allogeneic splenocytes derived from BALB/c mice (4 x 10^5^ cells/well) were co-cultured with BMDMs in 96-well plates for 72 hours in D10 medium (37°C, 5.5% CO_2_), at different Mφ:splenocyte ratios (1:2, 1:4, 1:8). To analyze the allogeneic T cell proliferation capacity, cell cultures were subsequently pulsed with [^3^H]-thymidine (1 μC/well; PerkinElmer, Germany) for additional 8-16 h. Culture supernatants were harvested onto Glass Fiber Filter Mates using an ICH-110 harvester (Inotech, Switzerland), and filters were counted in a 1450-microplate (Wallac, Finland). Cells of cocultures were also harvested after 72 h and used for flow cytometric analyses to determine frequencies of different T cell subsets.

### Phagocytosis assay

To analyze the ability of BMDMs to phagocytose and uptake *E.coli* bacteria, a gentamicin protection assay was performed. Bone marrow derived Mφ were generated from wt or cKO mice and seeded in 6 well plates in technical replicates in D10 medium without antibiotics. Cells were differentiated into CAM or AAM for 16h. Afterwards, Mφ were exposed to *E. coli* (DSM 1103) at an MOI = 10 for 1 hour. After rinsing the cultures three times in PBS to remove non-engulfed bacteria, cells were incubated for 1 h in fresh RPMI1640 medium containing 100 µg/ml of gentamicin to kill extracellular bacteria. Gentamicin was removed, and the cells were gently rinsed three times in PBS. BMDMs were lyzed by incubating them for three minutes in PBS containing 2 mM EDTA and 0.5% saponin, followed by transfer to Eppendorf tubes and high-speed vortexing for 30 s. Subsequently, cells were plated onto blood agar plates and the next day*, E.coli* colonies were counted.

### Western blot analyses

To assess CD83 protein content in whole cell lysates of murine BMDM, Western blot analyses were performed. In addition, protein levels of pSTAT6 and STAT6 in lysates of CD83wt or CD83-deficient Mφ were also analyzed by Western blotting. Thus, protein-lysates (20-30 µg per lane) were separated *via* SDS – polyacrylamide gel electrophoresis and blotted onto a nitro-cellulose membrane (GE Healthcare). After blocking in blocking reagent (5% BSA-TBST) membranes were incubated with the following primary antibodies overnight (4°C): goat CD83 (Clone: AF1437, R&D systems), mouse β-actin (Clone: AC-74, Sigma Aldrich), rabbit p-STAT6 (Clone: D8S9Y, Cell Signaling), rabbit STAT6 (Clone: D3H4, Cell signaling). Specific signals were detected using the appropriate HRP-labeled secondary antibody and the ECL Prime Western Blotting Detection Reagent (GE Healthcare). Quantification of Western Blots was performed using the ImageJ/Fiji software (48). The intensities of bands are visualized in bar graphs and represent the protein amount in arbitrary units. To analyze phosphorylation status of STAT6, band intensities of pSTAT6 were normalized to total STAT6 signals. β- Actin served as a loading control.

### RNA isolation

Total RNA was isolated from mock-, IFN-γ or IL-4-stimulated BMDMs generated from CD83wt or CD83 cKO mice. Cell pellets were lysed in RLT+β-Mercaptoethanol extracted by RNeasy Plus Mini Kit (Qiagen) according to the manufacturer’s instructions. In addition, wound biopsies were collected from CD83wt as well as cKO mice at day 0, day 3 or day 6, stored in RNAlater (Qiagen) at -80°C and subsequently used for further analyses. Homogenization of the tissue was performed in RLT+β mercaptoethanol using innuSPEED lysis Tube W (Analytic Jena). We performed three homogenization cycles à four minutes in the SpeedMill PLUS homogenizator (Analytic Jena).

### qPCR

Total RNA was reversely transcribed using the First strand cDNA synthesis Kit (Thermo Fisher Scientific GmbH), as described by the manufacturer. Briefly, 0.5-1 µg RNA was reversely transcribed and diluted 1:5 after synthesis. qPCR analyses were performed using the Sybr Green Super mix (Biozym) on a CFX96 Real time system (BioRad) and normalized to reference gene transcript *Hprt*. All primers were designed and validated according to the Minimum Information for Publication of Quantitative Real-Time PCR Experiments guidelines. For primer sequences, see **Table 1**.

**Table 1:** Primer sequences used in qPCR experiments (Sigma Aldrich).

Gene	Orientation	Sequences
*Cd83*	Forward Reverse	5’-CGCAGCTCTCCTATGCAGTG-3’ 5’-GTGTTTTGGATCGTCAGGGAATA-3’
*Cd200R*	Forward Reverse	5’-GGAGAACTTCTGCCCTAGCA-3’ 5’-AGTGTTCACTTGTGTCAGAGGA-3’
*Csf3*	Forward Reverse	5’-AGATCACCCAGAATCCATGG-3’ 5’-CCAGGGACTTAAGCAGGAAG-3’
*Gata3*	Forward Reverse	5’-CCAAGCGAAGGCTGTCGGCA-3’ 5’-TCCTCCAGCGCGTCATGCAC-3’
*Hprt*	Forward Reverse	5’-GTTGGATACAGGCCAGACTTTGTT-3’ 5’-GATTCAACTTGCGCTCATCTTAGGC-3’
*Il6*	Forward Reverse	5’-ACAAAGCCAGAGTCCTTCAGAG-3’ 5’-GAGCATTGGAAATTGGGGTAGG-3’
*Tnfa*	Forward Reverse	5’-GTGATCGGTCCCCAAAGGG-3’ 5’-CCAGCTGCTCCTCCACTTG-3’
*Cxcl1*	Forward Reverse	5’-ACTCAAGAATGGTCGCAAGG-3’ 5’-GTCCCATCAGAGCAGTCTGT-3’
*Msr-1*	Forward Reverse	5’-AGGTGTTAAAGGTGATCGGG-3’ 5’-ATCTTGATCCGCCTACACTC-3’
*Ym1/Chil3*	Forward Reverse	5’-GACTTGCGTGACTATGAAGC-3’ 5’-TGAATATCTGACGGTTCTGAGG-3’
*Tgfb*	Forward Reverse	5’-TGGAGCAACATGTGGAACTCTA-3’ 5’-AGACAGCCACTCAGGCGTATC-3’
*Acta-2*	Forward Reverse	5’-ATG CCT CTG GAC GTA CAA CTG-3’ 5’-CAC ACC ATC TCC AGA GTC CA-3’
*Col1a1*	Forward Reverse	5’-GAAGCACGTCTGGTTTGGA-3’ 5’-ACTCGAACGGGAATCCATC-3’

### Full-thickness excisional wound model and analyses of wound closure

To investigate the role of CD83 expressed by Mφ in physiological wound healing responses, we used the full-thickness excisional wound healing model as described previously (21). Briefly, CD83wt mice as well as CD83cKO mice were anesthetized using a mixture of ketamine and xylazine (120 mg/kg and 20 mg/kg body weight, respectively). In order to prevent wound healing by contraction and to give a defined scale for subsequent wound closure assessment, 8mm silicone rings (Thermo scientific) were mounted around the wound area, using vetbond (3M). Imaging was performed on day 0, 3, and 6 and wound diameters length (L) and width (W)) were determined by ImageJ and wound closure was calculated relative to the initial d0 wound dimension. Wound area (WA) on day X (dX) and wound closure (% of baseline) was calculated using the following equations:


Wound area (WA) = (L/2)∗(W/2)∗π

Wound closure (%) = (WA d0–WA dX)/WA d0∗100

On day 3 and 6 biopsies were obtained as 8 mm punches (pfm medicals) around the former wound area. Samples were either fixed in 4% paraformaldehyde for histological assessment or stored in RNAlater (Qiagen) at - 80°C for subsequent RNA analyses.

### Histology

For the histological assessment of wounds, skin biopsies were obtained on day 3 and day 6 after wound infliction using 8 mm biopsy punches around the wound area. Excised tissue was subsequently fixed in 4% paraformaldehyde and processed by conventional histological techniques, embedded in paraffin wax and sectioned at 5 µm thicknesses. Sections were mounted onto glass slides, de-paraffinized and stained with hematoxylin and eosin (HE).

### Statistical analyses

All statistical analyses were performed using GraphPad Prism 9.3.1 and the two-tailed unpaired student’s t- test or one- or two-way ANOVA for parametric data. Wherever necessary, we used non-parametric tests (Mann-Whitney-U or Kruskal-Wallis) when data was not normally distributed. Data are presented as mean values including the Standard Error Mean (SEM). P-values of *p<.05; **p<.01; ***p<.001; and ****p<.0001 were considered statistically significant.

## Data availability statement

The original contributions presented in the study are included in the article/**Supplementary Material**. Further inquiries can be directed to the corresponding author.

## Ethics statement

The animal study was reviewed and approved by Regierung von Unterfranken, Würzburg.

## Author contributions

KP-M designed, conducted and analyzed the majority of the experiments and prepared the manuscript. PL, AStr, LS, PM-Z, CK, and AW performed experiments, analyzed data and edited the manuscript. MW and JM provided scientific insights and helped with the Gentamicin protection assays. ASte, EZ, DR, and AW conceived and designed the study, supervised experiments and prepared the manuscript. All authors contributed to the article and approved the submitted version.
