# Supplementary material for: CD83 expressed by macrophages is an important immune checkpoint molecule for the resolution of inflammation
Source: Front Immunol. 2023 Feb 15;14:1085742. doi: 10.3389/fimmu.2023.1085742 (PMC9975560; doi:10.3389/fimmu.2023.1085742)
Supplement: Supplementary file 1 [file DataSheet_1.docx]

Supplementary Material:

Suppl. Fig. 1: Western Blot analyses to confirm KO of CD83 in BMDM generated from cKO mice. Full uncut gels. Western Blot analyses proof the presence of CD83 in unstimulated, IFN-γ-stimulated as well as IL-4 stimulated BMDM generated from CD83wt mice (lane 2-4, left WB). CD83 protein was undetectable in unstimulated BMDM as well as IFNγ- or IL-4 stimulated BMDM generated from cKO mice (lane 4-6, left WB). CD83 was detected using a anti-mouse CD83 specific monoclonal antibody conjugated to the specific secondary HRP-coupled antibody. β-Actin served as a loading control and was detected using a mAB against β-Actin conjugated to the specific secondary HRP-coupled antibody.

Suppl. Fig. 2: Gating strategy for murine BMDM generated from CD83wt and CD83 cKO mice. Bone-marrow derived Mφ were generated and differentiated in the presence of IFNγ- or IL-4 into CAM or AAM, respectively, for 16h. Subsequently, macrophages were analysed by flow cytometry. Doublets were excluded and the cell population was gated based on the forward and sideward scatter. Afterwards, dead cells were excluded (negative for 7-AAD) and living F4/80^+^CD11b^+^ Mϕ were selected for further analyses.

**Suppl. Fig. 3: Phenotype of BMDM generated from CD83wt or CD83 cKO mice.** Bone-marrow derived macrophages were generated from CD83wt or CD83 cKO mice and subsequently stimulated with IFN-γ or IL-4 for 16h. Unstimulated cells served as a control. Flow cytometric analyses revealed no differences between CD83wt or CD83-deficient macrophages regarding the expression of MerTK (**A**), CD206 (**B**), OX40L and CD40 (**C**) and PDL1, PDL2, PD1 (**D**).


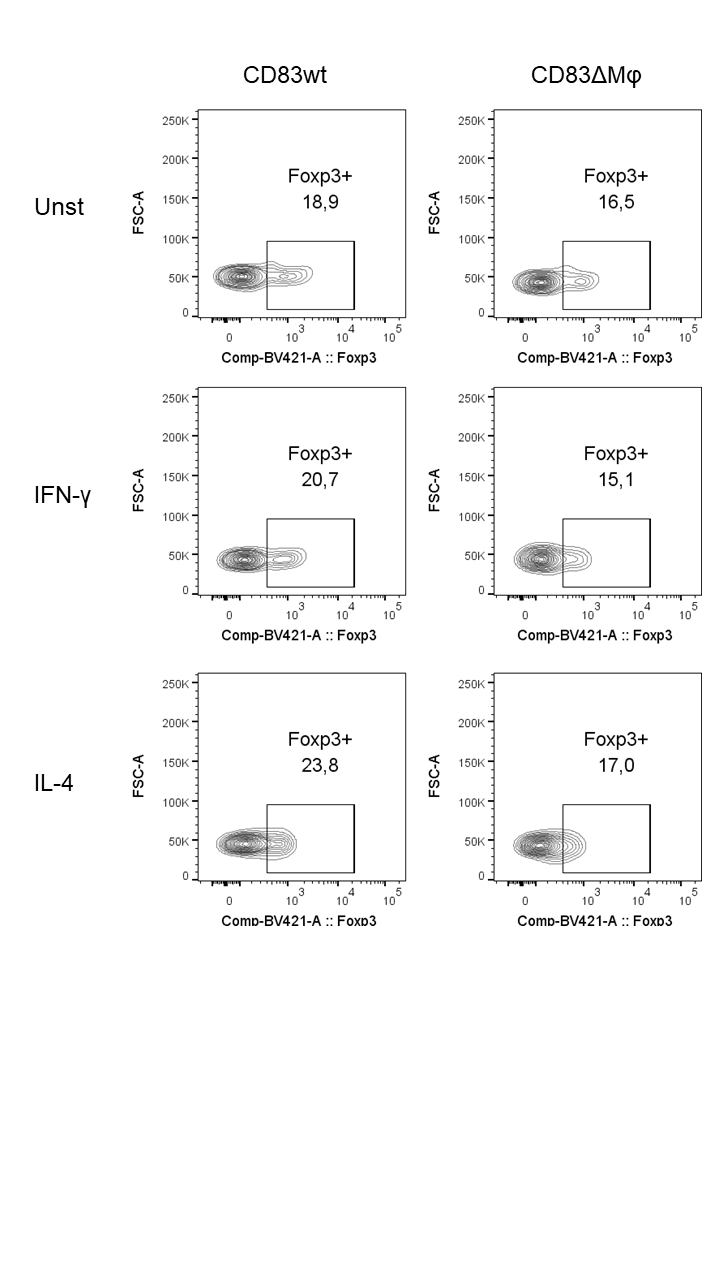


Suppl. Fig. 4: Representative FACS dot plots show the frequencies of CD4^+^Foxp3^+^ T cells in co-cultures of CD83wt (left hand side) or CD83ΔMφ (right hand side). Bone-marrow derived macrophages were generated from CD83wt or CD83 cKO mice and seeded in 96 well plates for stimulation with IFNγ (300U/ml) or IL-4 (40ng/ml), or left untreated. Subsequently, medium was discarded and single-cell suspension of Balb/C spleens were administered to Mφ. After 4 days we analyzed the composition of CD4^+^ T cell subsets by flow cytometry and representative FACS Dotplots for CD4^+^Foxp3^+^ T cells are displayed.
